# An Antibody Biosensor Establishes the Activation of the M_1_ Muscarinic Acetylcholine Receptor during Learning and Memory*^♦^

**DOI:** 10.1074/jbc.M115.681726

**Published:** 2016-01-29

**Authors:** Adrian J. Butcher, Sophie J. Bradley, Rudi Prihandoko, Simon M. Brooke, Adrian Mogg, Julie-Myrtille Bourgognon, Timothy Macedo-Hatch, Jennifer M. Edwards, Andrew R. Bottrill, R. A. John Challiss, Lisa M. Broad, Christian C. Felder, Andrew B. Tobin

**Affiliations:** From the ‡ Medical Research Council Toxicology Unit and; ¶ Protein and Nucleic Acid Chemistry Laboratory, University of Leicester, Hodgkin Building, Lancaster Road, Leicester LE1 9HN, United Kingdom,; § Eli Lilly and Co. Neuroscience, Erl Wood Manor, Windlesham, Surrey GU20 6PH, United Kingdom,; ** Eli Lilly and Co. Neuroscience, Lilly Corporate Center, Indianapolis, Indiana 46285, and; the ‖ Department of Molecular and Cell Biology, University of Leicester, Henry Wellcome Building, Lancaster Road, Leicester LE1 9HN, United Kingdom

**Keywords:** drug discovery, G protein-coupled receptor (GPCR), hippocampus, mass spectrometry (MS), phosphorylation, learning, memory

## Abstract

Establishing the *in vivo* activation status of G protein-coupled receptors would not only indicate physiological roles of G protein-coupled receptors but would also aid drug discovery by establishing drug/receptor engagement. Here, we develop a phospho-specific antibody-based biosensor to detect activation of the M_1_ muscarinic acetylcholine receptor (M_1_ mAChR) *in vitro* and *in vivo*. Mass spectrometry phosphoproteomics identified 14 sites of phosphorylation on the M_1_ mAChR. Phospho-specific antibodies to four of these sites established that serine at position 228 (Ser^228^) on the M_1_ mAChR showed extremely low levels of basal phosphorylation that were significantly up-regulated by orthosteric agonist stimulation. In addition, the M_1_ mAChR-positive allosteric modulator, 1-(4-methoxybenzyl)-4-oxo-1,4-dihydroquinoline-3-carboxylic acid, enhanced acetylcholine-mediated phosphorylation at Ser^228^. These data supported the hypothesis that phosphorylation at Ser^228^ was an indicator of M_1_ mAChR activation. This was further supported *in vivo* by the identification of phosphorylated Ser^228^ on the M_1_ mAChR in the hippocampus of mice following administration of the muscarinic ligands xanomeline and 1-(4-methoxybenzyl)-4-oxo-1,4-dihydroquinoline-3-carboxylic acid. Finally, Ser^228^ phosphorylation was seen to increase in the CA1 region of the hippocampus following memory acquisition, a response that correlated closely with up-regulation of CA1 neuronal activity. Thus, determining the phosphorylation status of the M_1_ mAChR at Ser^228^ not only provides a means of establishing receptor activation following drug treatment both *in vitro* and *in vivo* but also allows for the mapping of the activation status of the M_1_ mAChR in the hippocampus following memory acquisition thereby establishing a link between M_1_ mAChR activation and hippocampus-based memory and learning.

## Introduction

G protein-coupled receptors (GPCRs)^3^ respond to the binding of their cognate ligands by transitioning from an inactive to an active conformation capable of engaging with intracellular signaling cascades (1–3). Whereas this process has been described in exquisite pharmacological detail (4, 5), and biophysically in recent crystal structures (2, 6), correlating the activation state of a GPCR subtype *in vivo* to a physiological response or drug treatment is extremely challenging and presents a considerable barrier to establishing the physiological role of GPCRs and the on-target action of GPCR ligands.

Progress in this area has been made in transfected systems where it has been possible to monitor receptor conformational changes in response to ligand occupation using fluorescent resonance energy transfer (FRET). In these studies, changes in the energy transfer between FRET-acceptor and FRET-donor moieties, engineered within the receptor sequence, provide a read-out of changes in receptor conformation on agonist binding (7–10). Alternatively, a green fluorescent protein (GFP) biosensor based on a conformation-sensitive antibody (nanobody-80, Nb80) that preferentially recognizes the active state of the β_2_-adrenoreceptor has recently been employed to determine the active conformation of the β_2_-adrenoreceptor at the plasma membrane and within intracellular compartments (11). These approaches, however, require transfection of either mutated receptors (7–10) or a GFP biosensor (11), and therefore, although able to monitor receptor conformational changes quantitatively in living cells in real time, are restricted to heterologous systems.

An alternative approach considered here is to monitor the phosphorylation status of GPCRs as a read-out of receptor activation. This is based on the “classical” principle that the conformation adopted by a receptor upon agonist occupation reveals phosphorylation sites, often within the third intracellular loop and C-terminal tail, that otherwise are not accessible in the inactive receptor conformation (12). In this scenario, the phosphorylation status of a particular GPCR may serve as a read-out of the proportion of receptors that have adopted an active conformation. Therefore, phospho-specific antibodies to agonist-dependent receptor phosphorylation events could potentially be used as a probe for the activated receptor in not only recombinant systems, but also in physiologically relevant tissues. If this were the case, then it might be possible to correlate the activation status of GPCRs with physiological responses and, importantly for drug discovery, could be used to assess receptor engagement with synthetic ligands.

We test this notion here by focusing on the M_1_-muscarinic acetylcholine receptor (M_1_ mAChR), which is one of five muscarinic receptor subtypes (M_1_–M_5_) that respond to the natural ligand acetylcholine and is a subtype that has been implicated in a number of neurological processes (13, 14) most notably learning and memory (15–17). We have shown previously that this receptor subtype is rapidly phosphorylated by agonist addition likely via members of the G protein-coupled receptor kinase family (18), although the involvement of other receptor kinases has not been ruled out. Here, we used mass spectrometry-based phosphoproteomics to determine the sites of receptor phosphorylation from which we developed a series of phospho-specific antibodies. This included an antibody to phosphoserine 228 (Ser(P)^228^) in the third intracellular loop, which we show is a phosphorylation event highly sensitive to agonist stimulation. This antibody was used here to probe the phosphorylation status of the M_1_ mAChR following engagement with orthosteric and allosteric muscarinic ligands, both *in vitro* and *in vivo*. Furthermore, this antibody was used to determine that the M_1_ mAChR was activated in specific regions of the hippocampus following fear conditioning in a manner that maps to the regions of the hippocampus showing increased synaptic activity during memory acquisition. In this way we not only link the activation status of the M_1_ mAChR to memory acquisition but also establish the principle that phosphorylation sites can be used to probe the activation status of GPCRs during physiological responses and on drug treatment.

## Experimental Procedures

### 

#### 

##### Materials

Unless otherwise stated, all chemicals were purchased from Sigma. Xanomeline was obtained from Lilly (Erl Wood Manor, Windlesham, Surrey, UK).

##### Generation of Cell Lines

Chinese hamster ovary (CHO) cells that stably and constitutively expressed the C-terminal HA epitope-tagged mouse M_1_ mAChR (CHO-M1 cells) were generated using the Flp-In^TM^ system. CHO Flp-In cells were co-transfected with pcDNA5FRT containing M_1_ mAChR and pOG44, and transfected cells were selected with hygromycin B, and expression of M_1_ mAChR was confirmed by immunoblotting with anti-HA antibodies and by *N*-[^3^H]methylscopolamine (NMS) radioligand binding.

##### [^32^P]Orthophosphate Labeling and Immunoprecipitation

Cells were plated in 6-well plates at 200,000 cells/well 24 h before experimentation. For phosphorylation experiments, cells were washed three times with Krebs/HEPES buffer without phosphate (118 mm NaCl, 1.3 mm CaCl_2_, 4.3 mm KCl,1.17 MgSO_4_, 4.17 mm NaHCO_3_, 11.7 mm glucose, 10 mm HEPES (pH 7.4)) and incubated in this buffer containing 100 μCi/ml [^32^P]orthophosphate for 1 h at 37 °C. Cells were stimulated for 5 min with test compounds and immediately lysed by addition of buffer containing 20 mm Tris (pH 7.4), 150 mm NaCl, 3 mm EDTA, 1% Nonidet P-40, 0.5% sodium deoxycholate. The M_1_ mAChR was immunoprecipitated from the cleared lysates using anti-HA affinity matrix (Roche Applied Science). The washed immunoprecipitates were separated by SDS-PAGE on 8% gels that were then dried, and radioactive bands were revealed using autoradiography film. The films were scanned and bands were quantified using AlphaImager software (Alpha Innotech, San Leandro, CA).

##### M_1_ mAChR Purification and Mass Spectrometry

Stably transfected CHO-M1 cells expressing mouse HA-tagged M_1_ mAChR were grown until confluent in expanded surface rolling bottles at 0.25 rpm in a humidified CO_2_ incubator. For receptor purification, cells from four rolling bottles were harvested, resuspended in 40 ml of Krebs/HEPES buffer, and stimulated with 100 μm acetylcholine for 5 min. Membranes were then prepared and solubilized by addition of 5 ml of PBS containing 1% Nonidet P-40 plus a mixture of protease and phosphatase inhibitors (Complete, Roche Applied Science). After centrifugation at 20,000 × *g*, the resulting supernatant was diluted 1:1 with PBS, and the receptor was then purified on anti-HA affinity matrix. After extensive washing with solubilization buffer containing 0.5% Nonidet P-40, the resin was resuspended in 2× SDS-PAGE sample buffer. The sample was resolved by SDS-PAGE on 10% gels and stained with colloidal Coomassie Blue. The band associated with the M_1_ mAChRs was excised from the polyacrylamide and washed three times for 15 min with 100 mm triethylammonium bicarbonate (TEAB). Reduction and alkylation of cysteines were performed by addition of 10 mm dithiothreitol in 50 mm TEAB at 60 °C for 30 min followed by addition of 100 mm iodoacetamide in 50 mm TEAB for 30 min in the dark. Gel slices were washed three times for 5 min with 50 mm TEAB containing 50% acetonitrile, finally resuspended in TEAB containing 10% acetonitrile, and incubated overnight at 37 °C with 1 μg of sequencing grade trypsin (Promega, Southampton, UK). The resulting tryptic peptides were dried and resuspended in 1 ml of buffer containing 250 mm acetic acid and 30% acetonitrile, and phosphorylated peptides were enriched by addition of 20 μl of PHOS-Select^TM^ iron affinity resin and incubation at room temperature for 2 h with mixing. After washing the resin twice with loading buffer and once with water, tryptic phosphopeptides were eluted by addition of 200 μl of buffer containing 400 mm ammonium hydroxide and 30% acetonitrile. LC-MS/MS was carried out on each sample using an LTQ Orbitrap mass spectrometer (Thermo Fisher Scientific, Rockford, IL). Peptides resulting from in-gel digestion were loaded at a high flow rate onto a reverse-phase trapping column (0.3-mm inner diameter × 1 mm containing 5 μm C_18_ 300-Å Acclaim PepMap medium (Dionex, UK) and eluted through a reverse phase capillary column (75-μm inner diameter × 150 mm) containing Symmetry C_18_ 100-Å medium (Waters, Elsetree, UK) that was self-packed using a high pressure packing device (Proxeon Biosystems, Odense, Denmark). The resulting spectra were searched against the UniProtKB/Swiss-Prot database using Mascot (Matrix Science Ltd.) software with peptide tolerance set to 5 ppm and the MS/MS tolerance set to 0.6 Da. Fixed modifications were set as carbamidomethylcysteine with variable modifications of phosphoserine, phosphothreonine, phosphotyrosine, and oxidized methionine. The enzyme was set to trypsin/Pro, and up to two missed cleavages were allowed. Peptides with a Mascot score greater than 20 and for which the probability (*p*) that the observed match was a random event was <0.05 were included in the analysis. The spectra of peptides reported as being phosphorylated were interrogated manually to confirm the precise sites of phosphorylation.

##### Generation of M_1_ mAChR Antiserum and Phospho-specific M_1_ mAChR Antiserum

Phosphorylation-specific antibodies, anti-phosphoserine 228, 273, 322, and 451, were raised against peptide sequences AALQGpSETPGKG, RLLQAYpSWKEEE, KQPPKSpSPNTVK, and IPKRPGpSVHRTP corresponding to amino acid residues 223–234, 267–278, 316–327, and 445–456 of the mouse M_1_ mAChR. The 87-day program, which included four immunizations, was performed by Eurogentec. The resulting antisera were purified against the immunizing peptides. To generate antibodies for immunoprecipitation and detection of M_1_ AChR protein, rabbits and rats were immunized with the peptide RDRGGKGQKPRGKEQ that corresponds to amino acids 334–348 of the mouse M_1_ mAChR. The resulting antiserum was purified against the immunizing peptide.

##### Radioligand Binding Assays

CHO-M1 cell membranes (50 μg/tube) were incubated in HEPES buffer (50 mm HEPES, 110 mm NaCl, 5.4 mm KCl, 1.8 mm CaCl_2_, 1 mm MgSO_4_, 25 mm glucose, 58 mm sucrose (pH 7.4)) containing ∼0.3 nm [^3^H]NMS and increasing concentrations of acetylcholine in the presence of various concentrations of BQCA. Incubations were continued for 1 h at 37 °C. Nonspecific binding and filtration were carried out as above. Membrane-bound ligand was separated from free ligand by rapid filtration onto GF/B glass microfiber filters followed by three rapid washes with ice-cold 0.9% NaCl. Membrane bound radioactivity was determined by liquid scintillation (Ultima Gold, PerkinElmer Life Sciences) counting. Nonspecific binding was determined by the inclusion of atropine (1 μm) during the incubation with [^3^H]NMS.

##### M_1_ mAChR Immunoprecipitation from Tissue Samples

To establish the presence and phosphorylation status of M_1_ mAChRs in preparations from the hippocampus, tissues from adult wild-type or adult M_1_ mAChR knock-out mice on a C57Bl6/NTAC background were dissected into ice-cold Hanks' balanced salt solution buffer containing protease inhibitors and phosphatase inhibitors (Complete, Roche Diagnostics). Membranes were prepared and solubilized in buffer composed of 20 mm Tris (pH 7.4), 150 mm NaCl, 3 mm EDTA, and 1% Nonidet P-40. The receptor was immunoprecipitated with the anti M_1_ mAChR polyclonal antibody. Immune complexes were washed three times in solubilization buffer and resuspended in 2× SDS-PAGE sample buffer. Receptors were separated by SDS-PAGE on 8% gels, transferred PVDF membranes, and immunoblotted with anti-phosphoserine 228 antibodies or antibodies against the M_1_ mAChR protein.

##### Immunocytochemistry

CHO-M1 cells expressing mouse HA-tagged M_1_ AChR were seeded onto 20-mm glass coverslips for 24 h prior to experimentation. Cells were washed and incubated for 1 h in Krebs/HEPES buffer prior to treatment. Cells were fixed in PBS containing 4% paraformaldehyde and 0.1% glutaraldehyde for 30 min at room temperature. Anti-HA antibody was used at 50 ng/ml followed by Alexa Fluor^TM^ 546 goat anti rat secondary antibody at 1:1000. Anti-phospho-specific serine 228 antibodies were used at 0.25 μg/ml followed by goat anti-rabbit Alexa Fluor^TM^ 488 secondary antibody at 1:1000. Data were acquired using an Axiovert 200 M confocal laser scanning microscope (Zeiss).

##### Total [^3^H]Inositol Phosphate Accumulation Assay

Cells seeded at 100,000 cells/well in 24-well plates were labeled with 2.5 μCi/ml *myo*-[^3^H]inositol (PerkinElmer Life Sciences) for 24 h at 37 °C. Cells were washed twice in Krebs/HEPES buffer (pH 7.4) and incubated with 10 mm LiCl for 20 min at 37 °C. Appropriate concentrations of agonist were added for 20 min to stimulate [^3^H]inositol phosphate production. Where the allosteric interactions were studied, cells were pre-incubated with the allosteric modulator for 2 min prior to the addition of agonist. Incubations were terminated by aspiration of buffer and rapid addition of 500 μl of ice-cold trichloroacetic acid (TCA) (0.5 m). After extraction on ice for 20–30 min, samples were transferred to tubes containing 100 μl of EDTA (10 mm (pH 7.0)) and 500 μl of a 1:1 mixture of tri-*n*-octylamine, and 1,1,2-trichlorofluoroethane was added for 15 min at room temperature. After centrifugation at 20,000 × *g* for 2 min, 400 μl of the upper aqueous phase was transferred to fresh tubes containing 60 mm NaHCO_3_. [^3^H]inositol mono-, bis-, and trisphosphate ([^3^H]InsPx) fraction was recovered by anion-exchange chromatography on Dowex AG1-X8 formate columns. Columns were regenerated with 10 ml of ammonium formate (2 m)/formic acid (0.1 m) and washed thoroughly with distilled water. Samples were applied to the columns and the columns washed with 10 ml of distilled water. Columns were then washed with ammonium formate (60 mm)/sodium tetraborate (10 mm) solution. Total [^3^H]InsPx was eluted in 10 ml of ammonium formate (0.75 m)/formic acid (0.1 m) and collected in large scintillation vials. A 5-ml aliquot from the eluate was mixed with 10 ml of SafeFluor scintillation mixture, and radioactivity was detected by liquid scintillation counting.

##### Fear Conditioning Training

Male C57Bl6/NTAC mice (8–15 weeks old) were placed in the conditioning chamber (Stoelting ANY-maze fear conditioning system), and after a 2-min adaptation period, the mice received three tone/foot shock pairings, where a tone (conditioned stimulus, 2.8 kH, 85 db, 30 s) always co-terminated with the foot shock (unconditioned stimulus, 2 s, 0.4 mA). The conditioned stimulus-unconditioned stimulus pairings were separated by 1-min intervals. After completion of training, the mice remained in the conditioning chamber for 1 min and were returned to their home cages. For the immediate foot shock control, mice were put in the fear conditioning chamber, and a foot shock was immediately delivered (2 s, 0.4 mA); 30 s latter, they were returned to their home cages. Mice remained in their home cages for 30 min before being anesthetized with 3% isoflurane (2 liters/min O_2_) and transcardially perfused with 4% PFA. Following fixation, brains were immediately removed and further fixed overnight in 4% PFA.

##### Immunohistochemistry of Mouse Brain

Brains were processed in paraffin wax and sliced at 5 μm using a microtome. Following antigen retrieval, sections were washed in TBS containing 0.1% Triton X-100 and blocked for 2 h at room temperature in TBS, 0.1% Triton X-100, 10% goat serum, and 5% BSA. Sections were incubated with antibodies to c-Fos (Santa Cruz Biotechnology), ARC (Santa Cruz Biotechnology), and M_1_ AChR phospho-specific serine 228 at 2.5 μg/ml in blocking buffer (overnight at 4 °C). Sections were washed three times, and incubated with Alexa Fluor^TM^ 488 fluorescent secondary antibodies for 1 h at room temperature in blocking buffer. Following three washes, slices were mounted in Vectashield HardSet^TM^ mounting medium with DAPI. All images were taken using a Zeiss confocal microscope with Zen software (Zeiss).

##### Assay for ERK Activity

Extracellular signal-regulated protein kinase 1/2 phosphorylation assay in CHO-M1 cells were seeded into transparent 96-well plates at 35,000 cells/well and grown overnight at 37 °C. Cells were washed with PBS and incubated in serum-free α-minimal essential medium at 37 °C for at least 4 h. Cells were incubated with varying concentrations of agonist in the presence and absence of increasing concentrations of an allosteric compound for 5 min (compounds were co-added). The reaction was terminated by the removal of compounds and addition of 50 μl of SureFire lysis buffer. The lysates were agitated for 30 min at room temperature, and 4 μl of each lysate was transferred into a 384-well opaque Optiplate. SureFire detection mix (7 μl/well) consisting of detection reagent, activation reagent, donor beads, and acceptor beads (660:110:11:11 v/v) were added to the plate. Plates were incubated in the dark at room temperature for 2 h with gentle agitation before the fluorescence signal was measured using a PheraStar^TM^ plate reader.

##### Generation of M1 DREADD Mice

Transgenic C57BL6/J mice, which express a humanized, mutated form of the M_1_ mAChR, were generated by genOway (Lyon, France). Briefly, humanizing mutations V5A, S254T, K320R, G337A, and V413I were introduced into the mouse coding sequence to make the corresponding amino acid sequence identical to the human M_1_ mAChR. In addition, two point mutations, Y106A and A195G, were introduced that render the M_1_ mAChR insensitive to acetylcholine and promote sensitivity to the synthetic compound clozapine-*N*-oxide (CNO). This receptor mutant was termed the M1 DREADD receptor. Finally, an HA tag sequence YPYDVPDYA was appended to the C terminus followed by a stop codon. The gene-targeting construct consisted of long and short homology arms. The long arm consisted of a 5268-bp fragment containing the 3′ part of intron 2; this was generated by PCR from genomic DNA template using primers 85426GAlm 5′ GGGGACAACTTTGTATAGAAAAGTTGTTGGGAGGATGCAGCCTCTCCA and 85427GAlm 5′ GGGGACTGCTTTTTTGTACAAACTTGGGCGCGCCACGTACGCCTCTTCAAGGCTTAAGTGGAATGAAGGGGCAGCCCC. The short arm consisted of a 3188-bp fragment containing the 3′UTR sequence of exon 3 that was amplified from genomic DNA using primers 85424GAsm 5′ ACTGTCAACCCCATGTGCTACGCAC and 85425GAsm 5′ GGGCTACAAGGGAGCATGAACAAGC. A diphtheria toxin negative selection cassette was included to allow for selection of ES cells that had undergone homologous recombination, and a positive selection neomycin gene and a STOP of transcription cassette flanked by *lox*P sites were inserted upstream of the *CHRM1* coding sequence. This construct was transfected into C57BL6 ES cells that were selected using neomycin. Resistant colonies were screened to verify correct integration of the 5′ and 3′ homology arms by PCR using primers 85488 5′ GCAGGTCGAGGGACCTAATAACTTCG and 85489sa 5′ AAGCTCTCAAGGTCCAACAGTTTCTGG for short arm integration and primers 85487la 5′ AGTCTTACCAGGGAAGAAAGCCTGATCC and 0070-Neo-16219sa 5′ CCTGCTCTTTACTGAAGGCTCTTTACTATTGC for long arm integration.

Positive clones were expanded and injected into blastocysts derived from C57BL6/J female mice to generate chimeric animals that were then bred with C57BL6 wild-type C57BL6 mice to generate heterozygous F1 animals. The neomycin/STOP cassette was excised by breeding heterozygous animals with Cre deleter mice to generate M1 DREADD constitutive knock-in mice. Genotyping was performed by PCR on genomic DNA obtained from tail biopsies using primers mM1 451 forward 5′ TTG GTT TCC TTC GTT CTC TGG GC and mM1 1227 reverse 5′ GAC GTA GCA AAG CCA GTA GCC CAG C. The resulting PCR products were subjected to restriction digestion with SacI, the products of which discriminated between WT C57BL6 and heterozygous and homozygous M1 DREADD animals.

## Results

### 

#### 

##### Determination of the Phosphorylation Sites on the M_1_ mAChR

**FIGURE 1.**
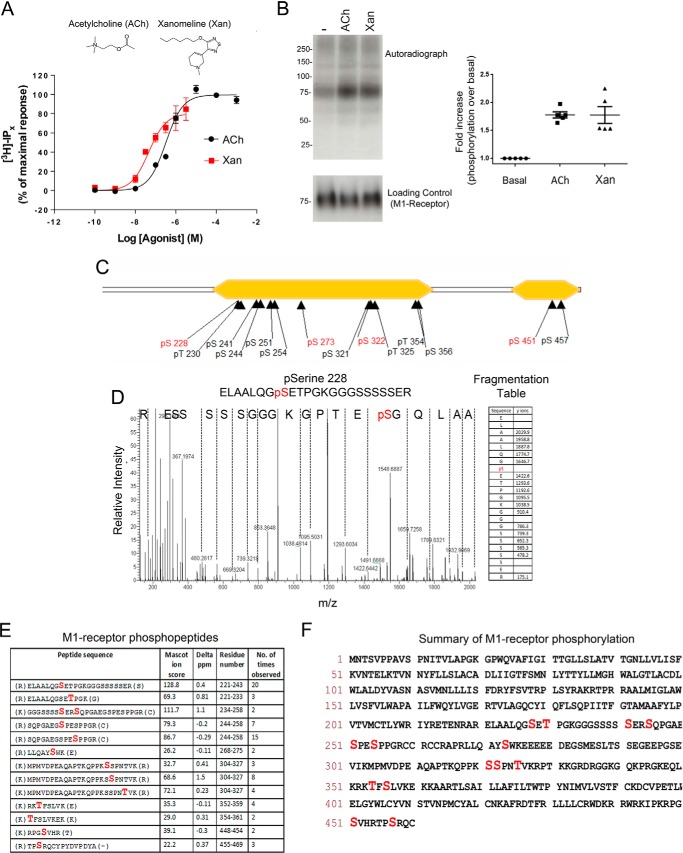
**Agonist-mediated phosphorylation of the M_1_ mAChR.** *A,* CHO cells expressing a C-terminally tagged mouse M_1_ mAChR (CHO-M1 cells) were treated with various concentrations of the muscarinic receptor agonists, acetylcholine (*ACh*) and xanomeline (*Xan*). Cells were then lysed and the inositol phosphate levels determined. Shown is the mean data of three experiments ± S.E. *B,* phosphorylation of the M_1_ mAChR was monitored in CHO-M1 cells metabolically labeled with [^32^P]orthophosphate and treated with vehicle, acetylcholine (100 μm), or xanomeline (*Xan*, 10 μm) followed by immunoprecipitation, gel electrophoresis, and autoradiography. Also shown are the mean data of five experiments ± S.E. The total M_1_ mAChR was also established using an anti-HA in a Western blot (*Loading Control*). *C,* summary of the mass spectrometric determination of the phosphorylation sites on the M_1_ mAChR. The phospho-sites indicated in *red* were the sites to which phospho-specific antibodies were raised. *D,* representative MS/MS spectrum and fragmentation table of M_1_ mAChR peptide phosphorylated on Ser^228^. *E,* summary of all the M_1_ mAChR phosphopeptides obtained from five experiments. Phosphorylated amino acids are highlighted in *red. F,* primary amino acid sequence of the human M_1_ mAChR receptor highlighting the phosphoacceptor sites in *red*.

Our initial evaluation of the phosphorylation status of the M_1_ mAChR was conducted on Chinese hamster ovary cells expressing the mouse M_1_ mAChR (CHO-M1 cells) stimulated with the natural ligand acetylcholine or the M_1_/M_4_-preferring agonist xanomeline (Fig. 1*A*) (19, 20). These two ligands stimulated inositol phosphate production in a concentration-dependent manner with acetylcholine and xanomeline both acting as full agonists (Fig. 1*A*).

In phosphorylation studies, where total receptor phosphorylation was determined using metabolically labeled CHO-M1 cells, both muscarinic agonists increased the phosphorylation status of M_1_ mAChRs (Fig. 1*B*). To establish the precise sites of phosphorylation, a mass spectrometry-based phosphoproteomic study was conducted that identified nine serine and three threonine phosphorylation sites in the third intracellular loop of the receptor, as well as two serine phosphorylation sites in the C-terminal tail (Fig. 1, *C–F*). Four of these sites at serine residues 228, 273, 322, and 451 (Ser^228^, Ser^273^, Ser^322^, and Ser^451^) were selected for the generation of phospho-specific antibodies based on the antigenicity of the sites.

**FIGURE 2.**
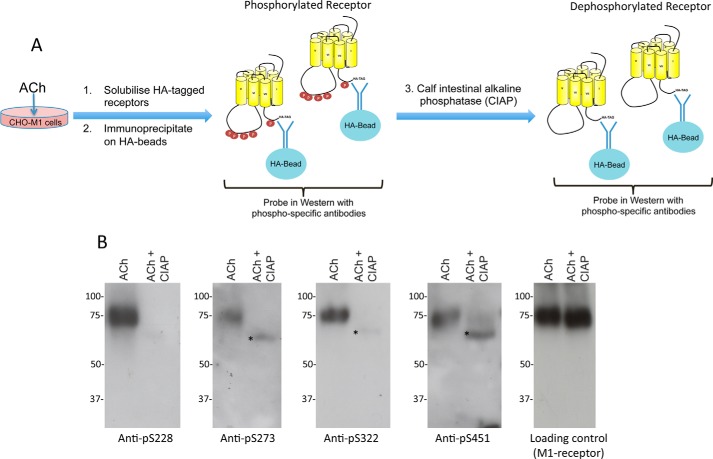
**Characterization of M_1_ mAChR phosphorylation-specific antibodies.** *A,* schematic representation of the experimental procedure used to characterize M_1_ mAChR phosphorylation-specific antibodies where CIAP was used to dephosphorylate immunoprecipitated M_1_ mAChR receptor derived from cells that had been treated with acetylcholine (*ACh*) before being probed in Western blots with the phospho-specific antibodies. *B,* results of Western blots from the experiments that are illustrated in *A*. Blots were probed with phospho-specific antibodies directed toward Ser(P)^228^, Ser(P)^273^, Ser(P)^322^, and Ser(P)^451^. Also shown is the loading control from this particular experiment using an anti-HA antibody to detect the epitope-tagged M_1_ mAChR. The * indicates slight cross-reactivity of the phospho-specific antibodies with CIAP.

The phospho-specific nature of the antibodies generated was tested by immunoprecipitation of M_1_ mAChRs from CHO-M1 cells (using antibodies that recognized the HA epitope engineered at the C terminus of the receptor) followed by treatment of the immunoprecipitate with calf intestinal alkaline phosphatase (CIAP) to remove the phosphate groups (Fig. 2*A*). Control immunoprecipitates were treated with vehicle. The immunoprecipitates were then probed in Western blots with the phospho-specific antibodies. In these experiments, treatment with CIAP removed the immunoreactivity for the M_1_ mAChR of all four phospho-specific antibodies (Fig. 2*B*) confirming that the antibodies were all phospho-specific.

##### Agonist-dependent M_1_ mAChR Phosphorylation

**FIGURE 3.**
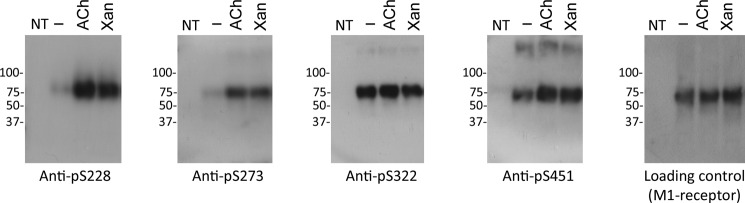
**Agonist-dependent phosphorylation revealed by phospho-specific antibodies to the M_1_ mAChR.** M_1_ mAChRs were immunoprecipitated from non-transfected (*NT*) CHO cells or CHO-M1 cells that were treated with vehicle, acetylcholine (*ACh*, 100 μm), or xanomeline (*Xan*, 10 μm) for 5 min, resolved by SDS-PAGE, and probed in Western blots with phospho-specific antibodies raised against Ser(P)^228^, Ser(P)^273^, Ser(P)^322^, and Ser(P)^451^. Total M_1_ mAChR was determined in the loading control using anti-HA antibodies. The experiment shown is typical of at least five independent experiments.

We next used the phospho-specific antibodies to determine whether phosphorylation on Ser^228^, Ser^273^, Ser^322^, and Ser^451^ was regulated by agonist stimulation. Probing lysates derived from CHO-M1 cells stimulated with vehicle, acetylcholine, or xanomeline in Western blots using the phospho-specific antibodies revealed that there were low levels of basal phosphorylation on residues Ser^228^ and Ser^273^ but that agonist treatment significantly increased phosphorylation at these residues (Fig. 3). This is in contrast to phosphorylation at Ser^451^ that showed notable basal phosphorylation that was only increased to a small extent in response to agonist treatment (Fig. 3). This was compared with phosphorylation at Ser^322^ that demonstrated high levels of basal phosphorylation and showed no substantive change in response to agonist (Fig. 3). In this respect, our panel of phospho-specific antibodies revealed that different phosphorylation sites on the M_1_ mAChR had different sensitivities to agonist stimulation.

Because the antibody used to detect phosphorylation at Ser^228^ gave a strong signal and phosphorylation at this site was exquisitely agonist-sensitive, this antibody was considered an excellent candidate for a biosensor that would identify the activated M_1_ mAChR receptor and as such was characterized further.

##### Determination of Changes in M_1_ mAChR Phosphorylation in Response to an Allosteric Ligand

**FIGURE 4.**
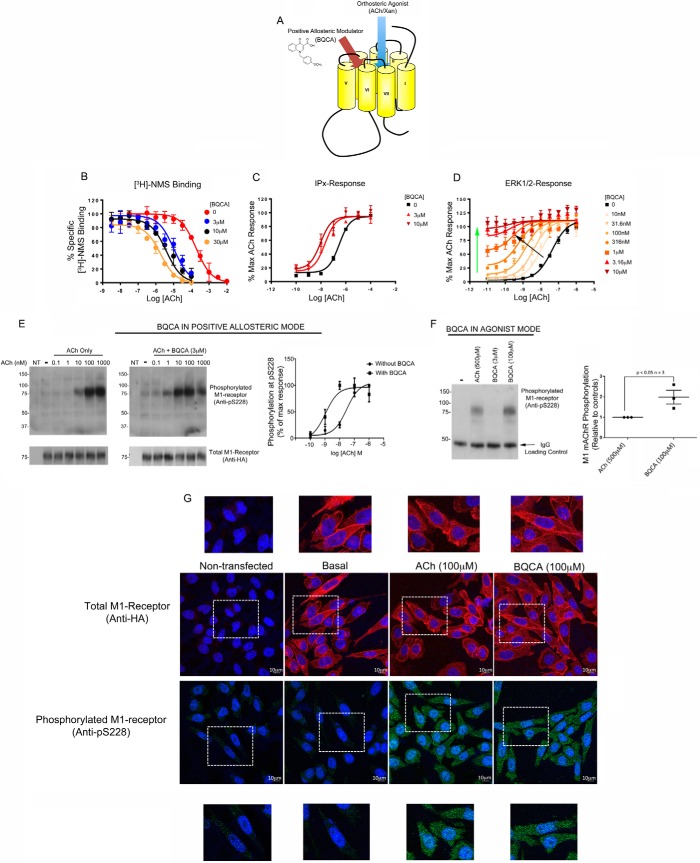
**Positive allosteric modulator (BQCA) promotes M_1_ mAChR phosphorylation at Ser^228^.** *A,* schematic representation of the M_1_ mAChR illustrating distinct binding modes for orthosteric ligands, acetylcholine (*ACh*) and xanomeline (*Xan*), from that of allosteric ligands, such as BQCA. The chemical structure of BQCA is also shown. *B,* CHO-M1 cell membranes were used in competition radioligand binding experiments where acetylcholine displacement of [^3^H]NMS was tested in the presence of a range of concentrations of BQCA. The data presented are means ± S.E. of three independent experiments conducted in triplicate. *C,* PAM activity of BQCA was revealed in concentration-response curves for acetylcholine-mediated inositol phosphate production in CHO-M1 cells in the presence of various concentrations of BQCA. *D,* both the mixed agonist (*green arrow*) and PAM (*black arrow*) activity of BQCA was revealed in concentration-response curves for acetylcholine pERK1/2 response in CHO-M1 cells in the presence of various concentrations of BQCA. *E,* representative Western blots of CHO-M1 cell lysates stimulated with increasing concentrations of acetylcholine in the presence or absence of BQCA (3 μm) and probed with the phospho-specific serine 228 antibody or anti-HA antibodies as a loading control for total M_1_ mAChR. Also shown are the mean data of three independent experiments ± S.E. *NT*, non-transfected cells. *F,* CHO-M1 cells were stimulated with a high concentration of acetylcholine (500 μm) or with BQCA at two concentrations; a low concentration (3 μm) where BQCA might be expected to show no intrinsic agonist activity and a high concentration (100 μm) where BQCA would show agonist activity. Cell lysates were prepared, resolved by SDS-PAGE, and Western blots probed with the phospho-specific serine 228 antibody. The IgG band was used as a loading control in these experiments. Also shown is the mean data ± S.E. of Western blots from three independent experiments. *G,* CHO-M1 cells stimulated with acetylcholine or BQCA were fixed and processed for immunocytochemistry using an anti-HA antibody to show total M_1_ mAChR or with the phospho-specific serine 228. Non-transfected CHO cells were similarly treated. All the images and gels shown were typical of at least three independent experiments. All the graphical data represent the mean ± S.E. of at least three independent experiments. Statistical analysis uses Student's paired *t* test.

The above data demonstrated that the activated state of the M_1_ mAChR in response to two orthosteric agonists (acetylcholine and xanomeline) could be established by monitoring the phosphorylation status of the receptor at Ser^228^. It is now well understood that the active conformation of GPCRs can also be promoted by ligands that bind at allosteric sites (Fig. 4*A*) (21, 22). Previous studies had established that the compound, 1-(4-methoxybenzyl)-4-oxo-1,4-dihydroquinoline-3-carboxylic acid (benzyl quinolone carboxylic acid; BQCA), is a selective positive allosteric modulator (PAM) to the M_1_ mAChR (23–25). We confirm these studies by establishing in our system that BQCA can promote acetylcholine binding at the M_1_ mAChR with a cooperativity factor of >100 (α factor = 128; Fig. 4*B*). The enhanced binding of acetylcholine observed in the presence of BQCA was correlated with an augmentation of acetylcholine signaling as demonstrated by a leftward shift in the InsPx concentration-response curve to acetylcholine when conducted in the presence of BQCA (cooperativity αβ factor = 85; Fig. 4*C*). Hence, BQCA was seen to promote the active conformation of the M_1_ mAChR, which was reflected in the ability of BQCA to augment agonist binding and agonist-mediated signaling.

Based on these observations, and given that phosphorylation at Ser^228^ likely reflects the proportion of M_1_ mAChRs in an agonist-occupied (R*) conformation, we reasoned that BQCA would promote acetylcholine-mediated phosphorylation at Ser^228^. This was indeed the case, with BQCA mediating an ∼100-fold shift in the potency of acetylcholine-mediated phosphorylation at Ser^228^ (pEC_50_ (−log *M*) for acetylcholine = 7.5 ± 0.1 and pEC_50_ for acetylcholine in the presence of BQCA (3 μm) = 8.9 ± 0.3; Fig. 4*E*). Thus, phosphorylation at Ser^228^ not only reflected the action of orthosteric ligands at the M_1_ mAChR (Fig. 3), but also reported the action of an allosteric modulator at this receptor.

These phosphorylation experiments were conducted with BQCA at a concentration that in itself gave no response (*i.e.* 3 μm), but rather it acted to enhance acetylcholine responses (*i.e.* a concentration where BQCA acted as a PAM). However, at higher concentrations, BQCA could be seen to act as an agonist in its own right (23, 25). The agonist properties of BQCA can be readily determined in pERK1/2 assays that, due to the high coupling efficiency between the M_1_ mAChR and pERK1/2 signaling in this system, were sufficiently sensitive to detect the agonist action of BQCA (Fig. 4*D*). Thus, high concentrations of BQCA in a pERK1/2 assay showed a direct agonist action (Fig. 4*D*). This agonist activity was reflected in complex concentration-response curves, where increasing concentrations of BQCA in the presence of very low concentrations of acetylcholine revealed the agonist activity of BQCA (*green arrow,* Fig. 4*D*), whereas higher concentrations of acetylcholine revealed mixed agonist and PAM activities of BQCA (*black arrow,* Fig. 4*D*). Importantly, when examined in phosphorylation studies, it was seen that at a high concentration (100 μm) BQCA mediated phosphorylation at Ser^228^ to a similar extent as that seen with a maximal concentration of acetylcholine (500 μm) (Fig. 4*F*). In contrast, a low concentration of BQCA (3 μm) alone did not stimulate any change in receptor phosphorylation (Fig. 4*F*).

Phosphorylation at Ser^228^ was further characterized in immunocytochemistry studies. In these studies, the Ser^228^ phospho-specific antibody detected very little immunoreactivity in non-transfected CHO cells and in CHO-M1 cells under basal conditions (Fig. 4*G*). However, following agonist treatment (acetylcholine, 5 min) or treatment with a high concentration of BQCA (100 μm), punctate cytoplasmic staining was detected (Fig. 4*G*). Interestingly, the punctate localization of the phosphorylated M_1_ mAChR represented only a small proportion of the total receptor population (identified using the antibodies against the HA epitope tag) that, after a 5-min stimulation, appeared concentrated at or very close to the plasma membrane (Fig. 4*G*).

By combining all these data, it can be concluded that muscarinic ligands that promote the transition of the receptor from an inactive R-state to an active R*-state promote phosphorylation of the M_1_ mAChR on Ser^228^. This includes orthosteric ligands, such as acetylcholine and xanomeline, as well as allosteric ligands (*i.e.* BQCA). In this sense, Ser^228^ phosphorylation is an ideal sensor of receptor activation, because this phosphorylation event reflects activation of the receptor regardless of the mechanism of activation.

##### Determination of the Active Conformation of the M_1_ mAChR during Memory Acquisition and Drug Treatment

**FIGURE 5.**
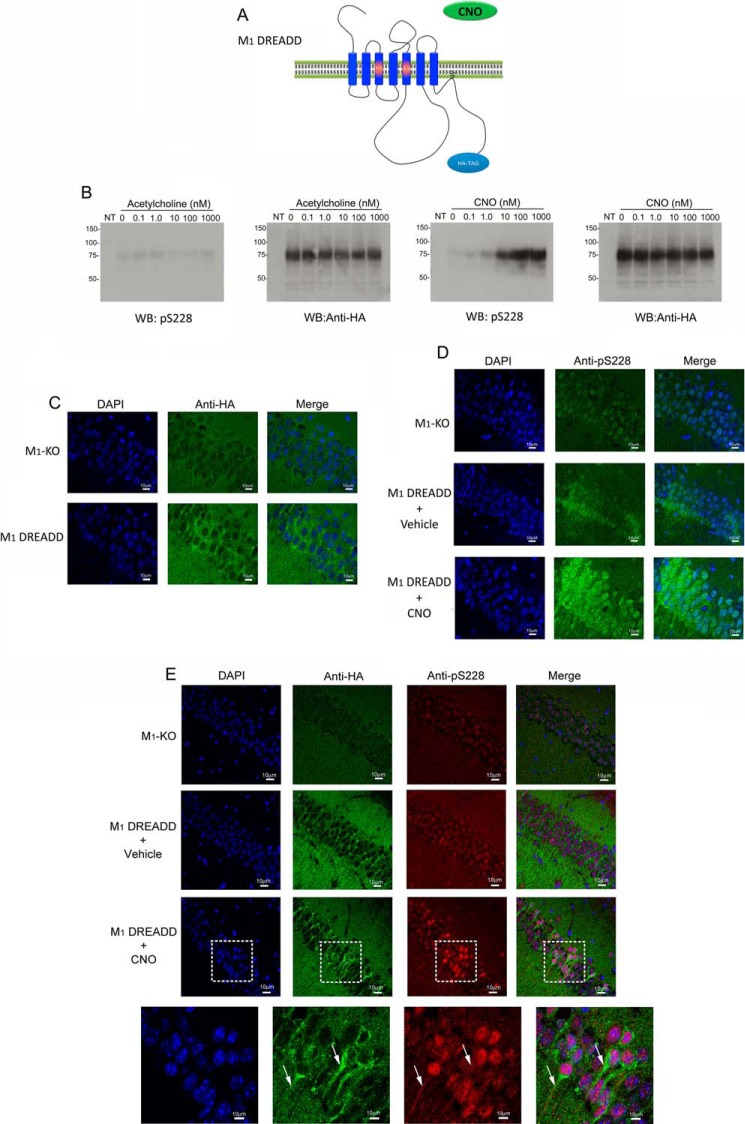
**Phosphorylation of Ser^228^ on the M1 DREADD receptor could be detected in the hippocampus following receptor activation with a selective agonist.** *A,* illustration of the two point mutations (Y106C and A195G) used to generation the HA epitope-tagged M1 DREADD receptor mutant in which activation by acetylcholine (*ACh*) is abolished but instead the receptor could be activated by CNO. *B,* CHO FlpIn cells expressing the HA-tagged M1 DREADD receptor were stimulated with increasing concentrations of acetylcholine or CNO. Western blots (*WB*) were probed with either anti-HA (as a loading control) or phospho-specific serine 228 antibodies. *NT*, non-transfected cells. *C* and *D,* M1 DREADD knock-in mice or M_1_ mAChR-knock-out mice (M1-KO) were injected (intraperitoneally) with CNO (0.3 mg/kg). After 30 min tissue was fixed by transcardial perfusion and sections stained with anti-HA antibodies (*C*) or phospho-specific serine 228 antibodies (*D*). *E,* fixed sections from M_1_ mAChR knock-out mice (*M1-KO*) or M1 DREADD knock-in mice treated with vehicle or CNO (0.3 mg/kg) were co-stained with anti-HA (*green*) and anti-phospho-specific serine 228 (*red*) antibodies. Two neurons where the staining for the receptor and the phosphorylated receptor occur in the same neuron are indicated by the *arrows*. The areas marked by the *white box* are magnified in the *lower panels*.

The data presented above support the notion that phosphorylation of Ser^228^ on the M_1_ mAChR can act as a biosensor for receptor activation in response to either orthosteric or allosteric ligands. To test whether this could be used to monitor the activation status of the M_1_ mAChR *in vivo*, we first employed a novel mouse model where the wild-type M_1_ mAChR gene was humanized so that the translated amino acid sequence was identical to the human M1 mAChR and where an HA epitope tag was inserted at the C terminus. Two further point mutations were introduced into the orthosteric binding site (Y106A and A195G) (Fig. 5*A*), which resulted in an HA-tagged humanized M_1_ mAChR mutant that was unable to be activated by the natural ligand acetylcholine but rather could be activated by the synthetic drug CNO (26, 27). This receptor mutant has been previously described and characterized *in vitro* and termed as a M1 DREADD (26). The design of this mouse means that expression of the M1 DREADD will be under the control of the native M_1_ mAChR promoter ensuring that the M1 DREADD would be expressed in the same cell types and at the same expression levels as the native M_1_ mAChR.

Analysis of the ability of acetylcholine and CNO to induce phosphorylation of the M1 DREADD was tested in transfected cells. It was found that acetylcholine had no significant effect on M1 DREADD phosphorylation at Ser^228^ (Fig. 5*B*). In contrast, the M1 DREADD was phosphorylated by CNO in a concentration-dependent manner (Fig. 5*B*). Immunohistochemical labeling of the HA epitope tag identified expression of the M1 DREADD mutant in the molecular layer of the CA1 region of the hippocampus (Fig. 5*C*). Using the Ser^228^ phospho-specific antibody in M1 DREADD identified light immunohistochemical staining in the CA1 region of the hippocampus that was up-regulated within 30 min of intraperitoneal administration of CNO (Fig. 5*D*).

Co-staining with anti-HA, to reveal the expression of the M1-DREADD HA-tagged receptor, and the Ser^228^ phospho-specific antibody revealed neurons where the M1-DREADD receptor existed in a phosphorylated state (indicated by *arrows* in Fig. 5*E*). Interestingly, not all of the M1-DREADD-expressing neurons stained with the phospho-specific Ser^228^ antibody suggesting that in some neurons the M1 receptor remained non-phosphorylated (at least at this residue) (Fig. 5*E*). It should be noted that M1-KO controls also revealed some weak nonspecific nuclear staining with the Ser^228^ phospho-specific antibody (Fig. 5, *D* and *E*).

**FIGURE 6.**
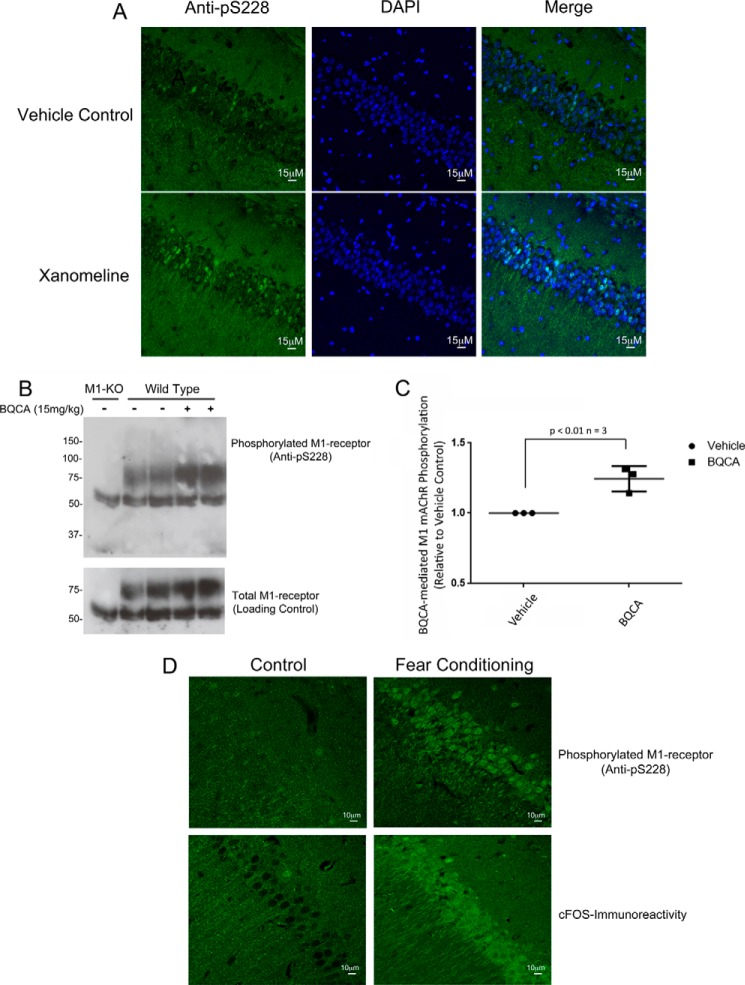
**M_1_ mAChR is activated in the hippocampus following drug treatment and memory acquisition.** *A,* C57/BL6/NTAC mice were injected (i.p.) with vehicle or xanomeline (5 mg/kg). After 30 min, tissues were fixed by transcardial perfusion, and sections were obtained and stained with the phospho-specific serine 228 antibody and with DAPI stain to reveal the nuclei. Shown are representative sections through the CA1 region of the hippocampus. *B,* C57/BL6/NTAC mice (*Wild-Type*) or M_1_ mAChR-knock-out mice (*M1-KO*) were injected (intraperitoneally) with BQCA (15 mg/kg) or vehicle, and after 30 min hippocampal membranes were prepared from which the M_1_ mAChR was immunoprecipitated. The sample was then processed in Western blots, which were probed with phospho-specific serine 228 antibody or an M_1_ mAChR-specific antibody to detect total M_1_ mAChR. *C,* quantification of Western blots from *B*. The data are presented as means ± S.E. (*n* = 3). Statistical analysis uses Student's paired *t* test. *D,* C57/BL6/NTAC mice were subjected to a fear conditioning training protocol or to an unpaired immediate foot shock as a control; 30 min later tissue was fixed by transcardial perfusion, and sections obtained and stained with phosphorylated Ser^228^-specific antibody (*upper panel*) or anti-c-FOS antibody (*lower panel*). All the data shown are typical of at least three independent experiments.

The data in the M1 DREADD mice supported the notion that the Ser^228^ phospho-specific antibody can be used to monitor M_1_ mAChR activation following stimulation by a synthetic ligand. We therefore tested this further by the administration of xanomeline (5 mg/kg i.p.) into wild-type mice followed by immunohistochemical analysis of phosphorylation at Ser^228^. Pharmacokinetic analysis determined that xanomeline levels in the brain peaked 30 min after injection. At this time the animal was sacrificed by perfusion fixation, and the brain was removed and sectioned for immunohistochemical staining. This procedure revealed phosphorylation of the M_1_ mAChR at Ser^228^ in the neuronal cell bodies in the pyramidal layer of the CA1 region of the hippocampus (Fig. 6*A*). In contrast, low levels of staining were observed in vehicle-treated mice (Fig. 6*A*).

To test whether phosphorylation at Ser^228^ could also act as a biosensor for M_1_ mAChR activation in response to the administration of a PAM, BQCA was administered at 15 mg/kg, which resulted in a free concentration of BQCA in the hippocampus of 51.8 ± 3.6 nm; a concentration where BQCA would show PAM activity at the M_1_ mAChR. In these experiments, Western blots of hippocampal lysates prepared from BQCA-treated animals showed an increase in the phosphorylation status of the M_1_ mAChR at Ser^228^ in response to BQCA (Fig. 6, *B* and *C*).

##### M1 mAChR Is Activated during Memory Acquisition

Having demonstrated that the action of an orthosteric agonist and a PAM at the M_1_ mAChR in the hippocampus could be monitored using the phosphorylation status of the M_1_ mAChR at Ser^228^, we next asked whether the phosphorylation of Ser^228^ could also be used as a biosensor for M_1_ mAChR activation during a physiological process. Previous studies had implicated a role for the M_1_ mAChR in learning and memory (15–17). Because the M_1_ mAChR is highly expressed in the hippocampus (28), an area associated with learning and memory, we asked whether changes in the phosphorylation status of Ser^228^ on M_1_ mAChRs could be detected in the hippocampus following fear conditioning training, a classical protocol used to induce a learning response (29). The mice were therefore subjected to fear conditioning training and 30 min were later sacrificed, tissues fixed by transcardial perfusion, and brains sectioned and probed with phospho-specific antibodies to Ser^228^ phosphorylation. In these experiments, an increase in the phosphorylation status of Ser^228^ was observed in the stratum pyramidale of the CA1 region of the hippocampus following fear conditioning training when compared with an immediate unpaired foot shock control (Fig. 6*D*). Importantly, this increase in Ser^228^ phosphorylation correlated with an increase in c-Fos immunoreactivity, a marker of neuronal activity (Fig. 6*D*).

## Discussion

Here, we show that phosphorylation at serine 228 in the third intracellular loop of the M_1_ mAChR can be used as a read-out for receptor activation. Using this antibody as a biosensor for the activated receptor, we show that the M_1_ mAChR is activated in the hippocampus following fear conditioning training. This correlates with an increase in neuronal activity in the hippocampus thereby strongly supporting a link between hippocampal M_1_ mAChR activity and hippocampus-based learning and memory (15–17). Our study also illustrates how analysis of GPCR phosphorylation status can be used to assess the activation of receptors following drug treatment. Establishing target-drug engagement is highly valued in drug discovery, but up to now, no method has been described to monitor this for a GPCR target.

To establish the phospho-specific antibody to Ser^228^ as a sensor for M_1_ mAChR activation, we first conducted a mass spectrometry-based phosphoproteomic analysis of the M_1_ mAChR expressed in a recombinant system. This revealed that the M_1_ mAChR, like many other GPCRs (30, 31), is multiply phosphorylated at sites within the third intracellular loop and C-terminal tail. The multisite nature of M_1_ mAChR phosphorylation is consistent with the suggestion made by us, and others, that the complex pattern of receptor phosphorylation is cell type-specific. This idea led to the notion that a receptor expressed in different cell types might show a different phosphorylation pattern, and this pattern would contribute to cell type-specific receptor signaling and regulation (30, 32, 33). This notion has been coined the receptor phosphorylation bar code (30, 32) and has been further extended to suggest that different agonists might drive different patterns of phosphorylation in a manner that encodes for different signaling outcomes (30, 32, 33). In this way, the phosphorylation bar code is thought to contribute to stimulus bias where a ligand can direct signaling down one pathway in preference to another (34, 35), possibly by mediating a specific pattern of receptor phosphorylation.

Given the notion that phosphorylation of GPCRs might be a dynamic process regulated, at least in part, by the pharmacological properties of the agonist used to stimulate the receptor, it was important in this study to carefully characterize the agonist dependence of the phosphorylation events revealed by the mass spectrometry studies. Hence, we generated a panel of phospho-specific antibodies to the M_1_ mAChRs that could be used to further probe at least 4 of the 14 sites of phosphorylation. First, all four of the antibodies generated were phospho-specific recognizing phosphorylation at Ser^228^, Ser^273^, Ser^322^, and Ser^451^. These antibodies established that there were high levels of constitutive phosphorylation at Ser^322^ and Ser^451^. In contrast, phosphorylation at Ser^228^ and Ser^273^ was seen to be highly sensitive to agonist stimulation and showed little phosphorylation in the basal state. Thus, phosphorylation at Ser^228^ and Ser^273^ appeared to fit the traditionally held view that only the agonist-occupied receptor undergoes phosphorylation (36, 37), and thus these sites were ideal candidates as sensors of the activated receptor.

Because of the high signal-to-noise ratio obtained with the phospho-specific antibody to phosphorylated Ser^228^, we used this in further studies and established that orthosteric agonists, acetylcholine and xanomeline, mediated phosphorylation of Ser^228^. Furthermore, the positive allosteric modulator, BQCA, was also able to drive Ser^228^ phosphorylation. Hence, it would appear that at least in the case of Ser^228^ phosphorylation, all ligands that promoted the transition of the receptor from an inactive R-conformation to an active R*-conformation resulted in phosphorylation of the receptor on Ser^228^ despite having different pharmacological properties. In this sense, an antibody that specifically recognized Ser^228^ phosphorylation could be considered as a biosensor of the active R*-conformation of M_1_ mAChR.

It is important to point out that not all phosphorylation sites on GPCRs will show the characteristics of Ser^228^ phosphorylation described here. Careful evaluation of the agonist sensitivity of the phosphorylation event with a range of pharmacological ligands would need to be conducted because other sites of phosphorylation might be less sensitive to agonist stimulation (such as Ser^322^ and Ser^451^) or might show different levels of phosphorylation in response to ligands. Such phosphorylation sites would not be suitable as indicators of receptor activation.

To determine whether the phospho-specific Ser^228^ antibody could be used to detect phosphorylation (and thereby activation) of the M_1_ mAChR *in vivo,* we employed a mutant mouse line that expressed a M1 DREADD receptor in place of the wild-type M1 mAChR. This receptor could be selectively activated by CNO *in vivo* (26). Importantly, our *in vitro* studies had established that CNO stimulated phosphorylation at Ser^228^ on the M1 DREADD. We show here that the M1 DREADD expressed in the CA1 region of the hippocampus was phosphorylated on Ser^228^ following administration of CNO. These data confirmed the hypothesis that the phospho-specific Ser^228^ antibody could be used to identify activated receptors in the hippocampus. This was further supported by receptor stimulation using xanomeline and BQCA in wild-type animals, where receptor activation could similarly be identified by an up-regulation of phosphorylation at Ser^228^.

Given that phosphorylation at Ser^228^ fit the criteria for use as a biosensor for receptor activation, we investigated whether the M_1_ mAChR was activated in the hippocampus during memory acquisition. Previous studies had established a role for muscarinic receptor signaling in learning and memory (14, 38, 39). The involvement of the M_1_ mAChR in this process is suspected not only because this receptor subtype is highly expressed in the hippocampus (28) but also because gene knock-out and pharmacological disruption of M_1_ mAChRs resulted in defective learning and memory (14). Thus, there is a great deal of interest in targeting the M_1_ mAChR as a mechanism to treat cognitive deficits in neurodegenerative disease, such as Alzheimer disease (14, 40, 41). Despite this, it is still unclear whether M_1_ mAChRs acting directly at the level of the hippocampus mediate memory processing or whether these receptors are more subtly involved by promoting interactions between the prefrontal cortex and hippocampus (16, 42–44). We were therefore interested to test the possibility that M_1_ mAChRs were activated in the hippocampus during memory acquisition. This was found to be the case with a significant up-regulation of M_1_ mAChR phosphorylation at Ser^228^ following fear conditioning training in the CA1 region of the hippocampus. Importantly, this region was also seen to show high levels of neuronal activity, as indicated by an increase in c-FOS expression, following fear conditioning training. These data support the hypothesis that hippocampal M_1_ mAChRs are activated during memory acquisition and that this may contribute to the process of learning and memory.

In conclusion, by determining the phosphorylation sites within the M_1_ mAChR and generating and characterizing the phospho-specific antibodies, we have established that at least one of the phosphorylation events (Ser^228^) is exquisitely sensitive to agonist stimulation. Using this phosphorylation event as a biosensor for receptor activation, we have been able to establish the activation of the M_1_ mAChR *in vivo* in the CA1 region of the hippocampus following drug treatment and memory acquisition.

## Author Contributions

A. J. B. conducted the primary experiments and assisted in writing the paper. S. J. B., R. P., S. M. B., A. M., J. M. B., T. M. H., J. M. E., and A. R. B. contributed to the experimental data. R. A. J. C., L. M. B., and C. C. F. contributed to experimental design and data analysis. A. B. T. conceived and led the study and wrote the paper.

## Footnotes

3 The abbreviations used are:

GPCR: G protein-coupled receptor
M_1_ mAChR: M_1_ muscarinic acetylcholine receptor
BQCA: 1-(4-methoxybenzyl)-4-oxo-1,4-dihydroquinoline-3-carboxylic acid
GRK: G protein-coupled receptor kinase
NMS: *N*-methylscopolamine
TEAB: triethylammonium bicarbonate
CNO: clozapine-*N*-oxide
DREADD: designer receptor exclusively activated by designer drug
InsPx: inositol phosphate
PAM: positive allosteric modulator
CIAP: calf intestinal alkaline phosphatase.
